# Potentiometric Determination of Ketotifen Fumarate in Pharmaceutical Preparations and Urine Using Carbon Paste and PVC Membrane Selective Electrodes

**DOI:** 10.1155/2011/604741

**Published:** 2011-10-13

**Authors:** Eman Y. Z. Frag, Gehad G. Mohamed, Mohamed M. Khalil, Mohammad M. A. Hwehy

**Affiliations:** ^1^Chemistry Department, Faculty of Science, Cairo University, Giza 12613, Egypt; ^2^Chemistry Department, Faculty of Science, Beni Suef University, Beni Suef, Egypt

## Abstract

This study compares between unmodified carbon paste (CPE; the paste has no ion pair) and polyvinyl chloride (PVC) membrane selective electrodes that were used in potentiometric determination of ketotifen fumarate (KTF), where sodium tetraphenylborate (NaTPB) was used as titrant. The performance characteristics of these sensors were evaluated according to IUPAC recommendations which reveal a fast, stable, and linear response for KTF over the concentration range of 10^−7^ to 10^−2^ mol L^−1^. The electrodes show Nernstian slope value of 52.51 ± 0.20 and 51.51 ± 0.25 mV decade^−1^ for CPE and PVC membrane electrodes at 30°C, respectively. The potential is nearly stable over the pH range 3.0–6.0 and 2.0–7.0 for CPE and PVC membrane electrodes, respectively. Selectivity coefficient values towards different inorganic cations, sugars, and amino acids reflect high selectivity of the prepared electrodes. The electrodes responses at different temperatures were also studied, and long operational lifetime of 12 and 5 weeks for CPE and PVC membrane electrodes, respectively, were found. These are used for determination of ketotifen fumarate using potentiometric titration, calibration, and standard addition methods in pure samples, its pharmaceutical preparations (Zaditen tablets), and biological fluid (urine). The direct potentiometric determination of KTF using the proposed sensors gave recoveries % of 98.97 ± 0.53 and 98.62 ± 0.74 with RSD 1.42 and 0.63% for CPE and PVC membrane selective electrodes, respectively. Validation of the method shows suitability of the proposed sensors for use in quality control assessment of KTF. The obtained results were in a good agreement with those obtained using the reported spectrophotometric method.

## 1. Introduction

**Figure 1:**
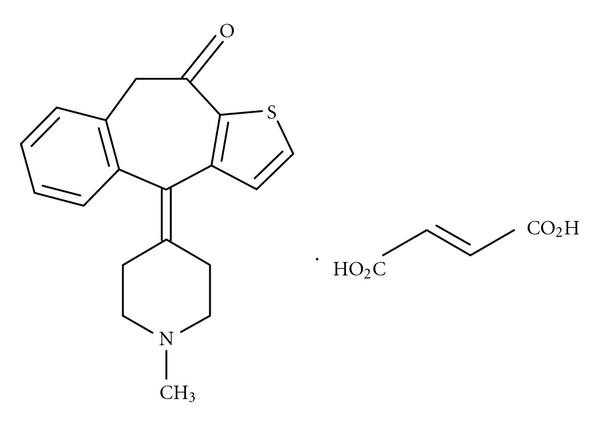
Structural formula of ketotifen fumarate.

Ketotifen fumarate (KTF) is designated chemically as 4-(1-methylpiperidin-4-ylidene)-4,9-dihydro-10*H*-benzo[4,5]cyclohepta[1,2-b]thiophen-10-one hydrogen(*E*)-butenediene. Its formula is C_19_H_19_NOS·C_4_H_4_O_4_, and its molecular mass: base: 425.5 g mol^−1^. It has the structure shown in Figure 1 [3, 4].

It is widely accepted as an antiasthmatic/antianaphylactic drug and also alleviates allergic disorders via a combination of several actions. For example, ketotifen is a relatively selective, noncompetitive antagonist of histamine H1 receptors and is a mast cell stabilizer, inhibiting the release of inflammatory mediators from mast cells [1, 2, 5]. Ketotifen fumarate was determined using spectrophotometric [6–10], chromatographic [11–15], and electroanalysis methods [16–19].

In recent years, the potentiometric membrane sensors have been widely used in pharmaceutical analysis [20–22]. This is mainly due to simple design, low cost, adequate selectivity, low detection limit, high accuracy, wide concentration range, and applicability of the selective electrodes to colored and turbid solutions. Potentiometric titrations were suitable for the determination of a relatively large amount of the drugs. The apparatus required for making potential measurements and performing titrations is generally inexpensive and basically simple in details. For this reason, the potential measurements find wide acceptance in industry as an analytical tool, both in the laboratory and in the process and quality control for routine analyses [23, 24].

This paper deals with the preparation of carbon paste (CPE) and polyvinyl chloride (PVC) membrane selective electrodes, and the performance characteristics of these sensors will be evaluated according to IUPAC recommendations [25]. The sensors will be used for the potentiometric determination of ketotifen fumarate in the pure form and in its pharmaceutical preparations and biological fluid (urine) using direct potentiometric, calibration, and standard addition methods. Detailed studies of the electrochemical behaviour of the electrodes were given.

## 2. Experimental Section

### 2.1. Reagents

All the reagents were of the analytical grade, and bidistilled water was used throughout the experiments. Ketotifen fumarate was supplied from Memphis Co. for Pharm. and Chem. Ind., Egypt. o-Nitrophenyloctylether (o-NPOE) from Fluka was used for the preparation of the sensors. Other types of plasticizers, namely, dibutylphthalate (DBP), dioctylphthalate (DOP), dioctylsebacate (DOS), and tricresylphosphate (TCP), were purchased from Merck, Sigma, Merck and Alfa-Aesar, respectively. Relative high molecular weight PVC was supplied from Aldrich. Ion-pairing agent, potassium tetraphenylborate (KTPB, Fluka), was used. Tetrahydrofuran (THF) was supplied from El-Nasr Company, Egypt.

### 2.2. Samples

Pharmaceutical preparations (Zaditen tablets, 1 mg/tablet) were provided by Novartis Pharma S.A.E., Cairo, Egypt.

### 2.3. Apparatus

Laboratory potential measurements were performed using 716 DMS Titrino Metrohm connected with 728 Metrohm stirrers. This Titrino had a combined electrode, which was more convenient to be used, equipped with silver-silver chloride double-junction reference electrode (Metrohm 6.0222.100) in conjugation with different surfactant ion-selective electrodes. Microanalyses for carbon, hydrogen, nitrogen, and sulphur were carried out at the Microanalytical centers, Cairo University, using a Perkin-Elmer CHN 2400 elemental analyzer. pH measurements were carried out with a Jenway pH-meter model 3505.

### 2.4. Electrode Preparation

Carbon paste electrode was prepared by hand mixing accurately weight (500 mg) of highly pure graphite powder and plasticizer (0.2 mL of DOP, TCP, DBP, DOS, or *o-*NPOE) using an agate mortar where the paste mixture was packed into a piston driven Teflon holder [26]. The fabricated CPE was conditioned in distilled water for 24 h and soaked in freshly prepared ion pair suspensions.

For PVC electrode, the cocktail (consisting of 240 mg *o-*NPOE, 240 mg PVC, and 6 mL THF) was stirred for 5 min and poured into Petri dish “5 cm” diameter. After 24 h of slow evaporation of solvent, a master membrane with 0.11 mm thickness was obtained which was mounted on the softened end of the PVC tubing with the help of adhesive solution prepared by dissolving PVC in THF. The PVC closed tube with the membrane was filled with 0.25 mL of 1 mol L^−1^ KCl and completed to 25 mL by 1.0 × 10^−2^ mol L^−1^ KTF drug solution under investigation using Ag/AgCl as internal reference electrode. The fabricated electrodes were soaked in ion pair solution for 24 hr.

### 2.5. Effect of Temperature on the Electrode Response

The potential response displayed by the CPE and PVC membrane electrodes was monitored as a function of temperature in the range of 10–40 and 10–60°C for CPE and PVC sensors, respectively, for 5 minutes at 10°C degree interval using KTF concentration of 10^−2^ mol L^−1^.

### 2.6. Effect of pH on the Electrode Response

The effect of pH on the potential values of the two electrode systems was studied over the pH range 2–12 at 1-pH unit interval. Each electrode was immersed in 10^−2^ and 10^−4^ mol L^−1^ KTF solutions. The pH values were recorded, while aliquots of diluted sodium hydroxide or hydrochloric acid solutions were added.

### 2.7. Calibration of Electrodes

The new CPE and PVC sensors were calibrated by transferring 3 mL aliquots of 10^−7^ to 10^−2^ mol L^−1^ KTF solutions into 25 mL beaker at 25°C followed by immersing the ISE for each KTF in conjugation with Ag/AgCl reference electrode in the solution. The potential change was plotted against the logarithm of KTF concentration from which the calibration curve was constructed.

### 2.8. Analysis of Pharmaceutical Sample

Potentiometric determination of KTF in pharmaceutical samples. A known volume of Zaditen tablets (1 mg/tablet) was made up to 25 mL with water in a volumetric flask and filtered. 3 mL aliquot of the dilute solution was transferred to a 25 mL beaker. The content of KTF in the pharmaceutical preparations was estimated via potentiometric titration with KTPB.

### 2.9. Urine Sample Preparation

A urine sample was obtained from a healthy volunteer and spiked with 4.0 × 10^−7^ g L^−1^ KTF standard solution. The synthetic urine sample was centrifugated at 2500 rpm for 10 min. Then, the top layer was separated then directly analyzed using the proposed sensors.

## 3. Results and Discussion

The KT-TPB ion pair is formed in 1 : 1 [KT^+^] :  [TPB^−^] ratio, and it has white colour and is characterized using elemental analysis with calculated %C = 82.30, %H = 6.22, %N = 2.23, and %S = 5.10, and found %C = 79.53, %H = 7.06, %N = 2.05, and %S = 5.70. This finding is consistent with the previously published data [17].

### 3.1. Calibration of the Electrode

**Figure 2:**
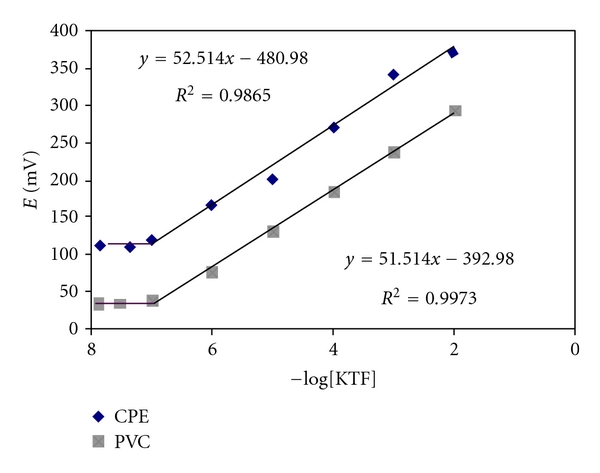
Calibration curve for KTF using CPE and PVC membrane electrodes.

The CPE and PVC sensors were calibrated by immersing the electrode plasticized with DBP in conjunction with the double junction Ag/AgCl reference electrode in solutions of KTF in the range of 10^−2^–10^−7^ mol L^−1^. They were allowed to equilibrate whilst stirring and recording the e.m.f. readings. The CPE and PVC membrane sensors showed a linear response over the concentration range from 10^−7^–10^−2^ mol L^−1^ with Nernstian slope of 52.51 ± 0.20 and 51.51 ± 0.25 mV decade^−1^ and detection limit of 9.81 × 10^−8^ and 1.20 × 10^−7^ for CPE and PVC membrane electrodes, respectively, (Figure 2).

### 3.2. Effect of Plasticizer

Five plasticizers, DOS, o-NPOE, DOP, TCP, and DBP were used to examine the optimization of the electrode with plasticizer. The results obtained showed that the response performances of the membranes prepared were rather different depending on the use of plasticizer. The best plasticizer was found to be o-NPOE and DOS for CPE and PVC membrane electrodes. 

The analytical performance of CPE is compared with the PVC membrane electrode using o-NPOE and DOS, respectively. The CPE has the best performance with respect to total potential change, potential break at the end point, as well as the response time in comparison with PVC electrode.

### 3.3. Effect of Soaking Time

Freshly prepared electrodes must be soaked to activate the surface of the carbon paste and PVC membrane layers to form an infinitesimally thin gel layer at which ion exchange occurs. This preconditioning process requires different times depending on diffusion and equilibration at the electrode-test solution interface; a fast establishment of equilibrium is certainly a condition for a fast potential response. Thus, the performance characteristics of the KTF ion-selective electrodes were investigated as a function of soaking time. For this purpose, the CPE and PVC membrane electrodes were soaked in KTF-TPB ion-pair suspension and the titration curves were plotted from which the total potential changes are recorded after 0, 15, 30, 60, 120 min and 12 and 24 hr. The optimum soaking time was found to be 5 and 30 min for CPE and PVC membrane electrodes, respectively.

### 3.4. Effect of pH

**Figure 3:**
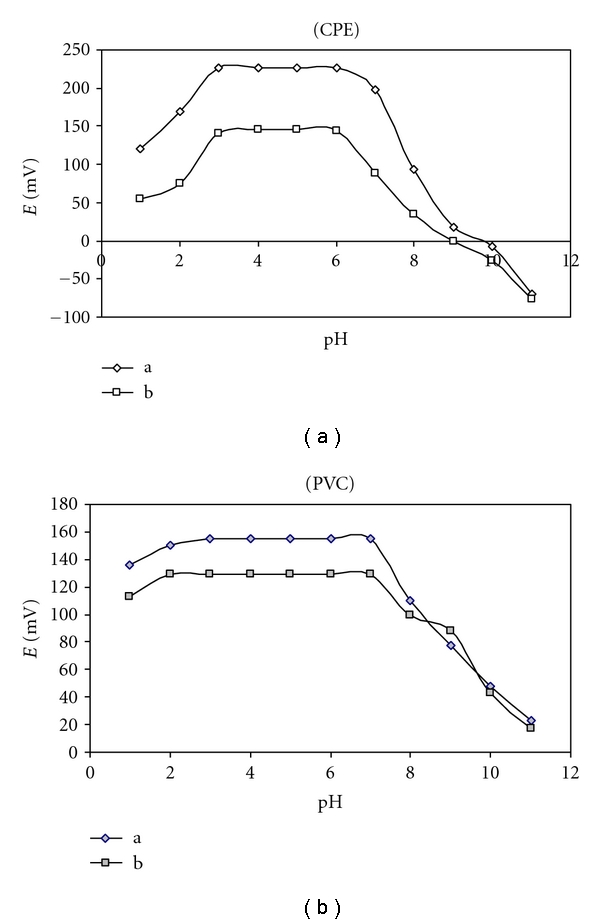
Effect of pH on (a) 10^−2^ and (b) 10^−4^ mol L^−1^ of KTF on the potential readings of CPE and PVC membrane electrodes.

The influence of pH on the response of the CPE and PVC membrane sensors was checked by recording the potential readings of the cell for solutions containing 10^−4^ and 10^−2^ mol L^−1^ of KTF at different pH values (pH 2–11). Variation of pH value was done by adding very small volumes of HCl and/or NaOH solution (0.1–1 mol L^−1^ of each) to 5 mL of the KTF solution and plotting E (mV) versus pH values (Figure 3). The plots of E (mV) versus pH indicate that the response of the electrodes was pH independent in the pH range 3.0–6.0 and 2.0–7.0 for CPE and PVC electrodes, respectively. At pH value less than 2, the potential increases which may be due to the formation of protonated species, while at pH value higher than 6 or 7, the potential decreases, this may be due to the deprotonation of KTF drug.

### 3.5. Selectivity of the Electrode

The selectivity coefficients (log⁡⁡*K*_*D*,*B*_^pot^) for some inorganic cations of the CPE and PVC membrane were determined employing separate solution method (SSM) with the rearranged Nicolsky equation [27, 28]:

(1) $$\log K_{D,B}^{\text{pot}}=\left(\frac{E_{1}-E_{2}}{S}\right)+\left(1+\frac{z_{1}}{z_{2}}\right)\log a,$$

where, *E*_1_ is the potential measured in 1 × 10^−3^ mol L^−1^ KTF (*D*), *E*_2_ the potential measured in 1 × 10^−3^ mol L^−1^ of the interfering compound (*B*), *z*_1_ and *z*_2_ are the charges of the KTF (*D*) and interfering species (*B*), respectively and *S* is slope of the electrode calibration plot. While the selectivity coefficients for of many nitrogenous compounds such as starch, sugars and glycine was obtained by the matched method which is totally independent of the Nicolsky equation:

(2) $$\log K_{D,B}^{\text{pot}}=\frac{\left(a_{D}^{\prime }-a_{D}\right)}{a_{B}}.$$

To determine the selectivity coefficients by the matched method a known activity (*a*_*D*_′) of the primary ion solution is added into a reference solution that contains a fixed activity (*a*_*D*_) of primary ions, and the corresponding potential change (Δ*E*) is recorded. Next, a solution of interfering specie is added to the reference solution until the same potential change (Δ*E*) is reached and the activity of interfering (*a*_*B*_) is recorded. The change in potential produced at the constant background of the primary ion must be the same in both cases. The results obtained are summarized in Table 1.

The influence of some inorganic cations, sugars, and glycine on the KTF-electrodes was investigated. The selectivity coefficients values of the CPE and PVC membrane electrodes reflect a very high selectivity of the investigated electrodes for the ketotifen cation (KT^+^). The inorganic cations do not interfere owing to the differences in ionic size, and consequently their mobilities and permeability, as compared with those of KT^+^. Also, the smaller the energy of hydration of the cation, the greater the response of the membrane. In the case of sugars and glycine, the high selectivity is mainly attributed to the difference in polarity and lipophilic character of their molecules relative to KTF [24]. 

### 3.6. Effect of Temperature

**Figure 4:**
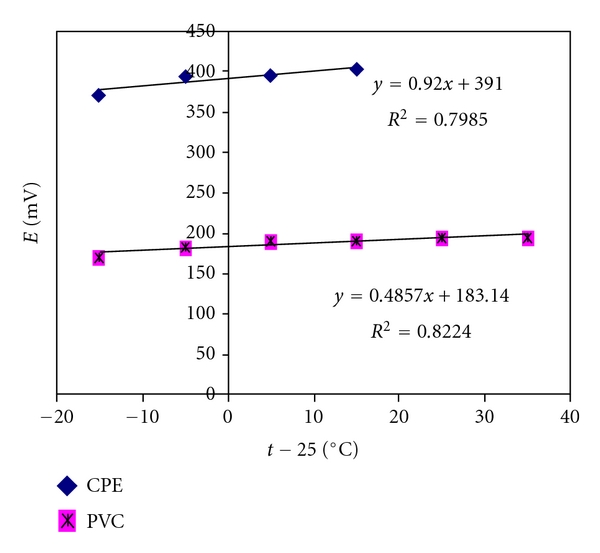
Effect of temperature CPE and PVC membrane electrodes.

To study the effect of temperature, the electrode potential of 10^−3^ mol L^−1^ KTF solutions were determined in 10, 20, 30, 40, 50, and 60°C and the standard electrode potentials (*E*°_elec._) (obtained from the calibration plots as the intercepts at pKTF = 0) corresponding to each temperature is determined. For the determination of the isothermal coefficient (d*E*°/d*T*) of the electrodes, the standard electrode potential (*E*°_elec._) at different temperatures was plotted versus (*t* − 25), where *t* is the temperature of the test solution (Figure 4). A straight-line plot was obtained according to the following [24]:

(3) $$E^{{}^{\circ}}={E^{{}^{\circ}}}_{\left(25\right)}+\left(\frac{\text{d}E^{{}^{\circ}}}{\text{d}T}\right)\left(t-25\right).$$

The slope of the straight line obtained (*E*° = −122.07 + 2.701(*t* − 25)) represents the isothermal coefficient of the electrode (amounting to 0.00092 and 0.00049 V/°C for CPE and PVC sensors, resp.) and reveals a good thermal stability of the electrode within the permitted temperature range.

### 3.7. Life Time

For the determination of the storage stability, the fabrication electrodes were tested weekly in the potentiometric titration of 10^−2^ mol L^−1^ KTF by using 10^−2^ mol L^−1^ NaTPB. Carbon paste electrode was found to be more stable than PVC selective membrane electrode and long operational lifetime (12 and 5 weeks for CPE and PVC, resp.) as shown in Table 2.

### 3.8. Potentiometric Determination of Ketotifen Fumarate in Pure, Pharmaceutical Preparations, and Urine

The response characteristics of CPE and PVC sensors are given in Table 2. In pharmaceutical analysis, it is important to test the selectivity toward the excipients and the fillers added to the pharmaceutical preparations. Fortunately, such materials mostly do not interfere. This is clear from the results obtained for the pharmaceutical preparations (Table 3) that these excipients do not interfere.

**Figure 5:**
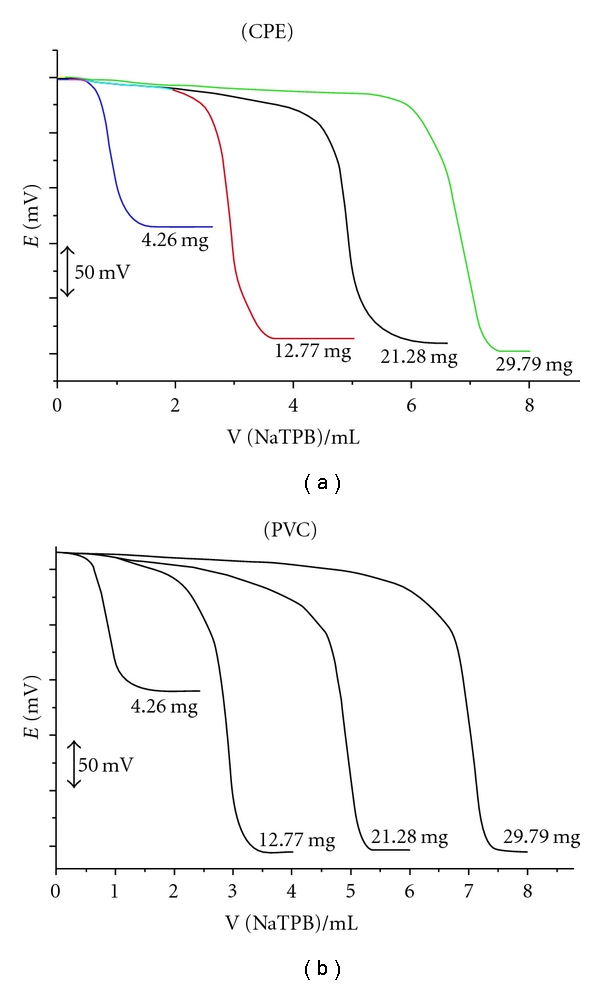
Potentiometric titration of KTF against 10^−2^ mol L^−1^ NaTPB using CPE and PVC membrane electrodes.

The electrodes were used as a sensor for determination of different concentrations of KTF (4.255–29.785 mg) in pure solutions (Figure 5) applying the potentiometric titrations and standard addition method, and the recovery % and RSD% were listed in Table 3. The mean % recovery and RSD% indicate that the validated method could be adopted for the determination of the investigated drug in its pharmaceutical preparations without interference from the coformulated adjuvants. The results obtained applying the CPE and PVC membrane sensors have the advantage that it does not need any extraction or separation. 

Direct, calibration, and standard addition techniques were utilized for the determination of KTF drug in spiked urine samples. The mean recoveries obtained were given in Table 4. The proposed electrodes can therefore be applied for determination of KTF in pure form, pharmaceutical formulations, and in urine samples without fear of interference caused by the excipients expected to be present in tablets or in the constituents of body fluids.

## 4. Validation of the Proposed ISE Method

### 4.1. Accuracy

For the determination of ketotifen fumarate in pure solutions and pharmaceutical preparations, the accuracy of the proposed ISE method (using CPE and PVC membrane electrodes) was investigated. It is clear from the results summarized in Table 3 that the proposed CPE and PVC ISE method is an accurate one for the determination of ketotifen fumarate in its pharmaceutical preparations without interferences from the coformulated excipients as indicated by the percentage recovery values.

### 4.2. Linearity

Under the optimal experimental conditions, linear relationships exist between the electrode potential/mV and the logarithm of corresponding concentration of the drug (Figure 2). The regression data, correlation coefficients (*r*), and other statistical parameter are previously listed in Table 2.

### 4.3. Precision

The precision of the proposed CPE and PVC membrane method, measured as percentage relative standard deviation (RSD%), was tested by repeating the proposed method for determination of the investigated drug in its pharmaceutical preparations of three replicates. The RSD% values for the repeated determinations were found to be 1.54 and 1.69% for CPE and PVC membrane selective electrodes, respectively, (Table 3). The RSD values are less than 2% indicating a good precision.

## 5. Conclusion

The present work demonstrates the fabrication of CPE and PVC electrodes utilizing different preparation methods. The proposed electrodes showed Nernstian slopes in the concentration range 10^−7^ to 10^−2^ mol L^−1^. The fabricated electrodes were successfully applied for the potentiometric determination of KTF in pure, pharmaceutical forms and biological fluids. Additionally, the proposed method has some important advantages: the electrode proved to be successful, providing a rapid, simple, and low cost potentiometric method for the determination of ketotifen fumarate in pure solutions, in pharmaceutical preparations, and in urine. It ensures a good accuracy for the ketotifen fumarate assay due to the possibility to control the ion activity continuously and also a fast assay of ketotifen tablets.

## Figures and Tables

**Table 1:** Potentiometric selectivity coefficient of CPE (DOS) and PVC (DOS) membrane electrodes.

Interfering ions (B)	log⁡⁡*K*_*D*,*B*_^pot^			
	SSM		Matched method	
	CPE	PVC	CPE	PVC
Glucose	—	—	3.95	2.20
Lactose	—	—	3.90	4.13
Fructose	—	—	2.85	1.60
Maltose	—	—	3.65	0.35
Starch	—	—	2.50	1.50
Sucrose	—	—	4.00	1.10
Glycine	—	—	2.30	1.15
Ca^2+^	1.30	1.70	—	—
Ni^2+^	1.70	1.20	—	—
Co^2+^	2.40	0.60	—	—
Cd^2+^	2.50	0.90	—	—
Na^+^	3.00	1.09	—	—

**Table 2:** Critical response characteristics of CPE and PVC sensors.

Parameter	CPE	PVC
Linear range (mol L^−1^)	10^−7^ to 10^−2^	10^−7^ to 10^−2^
Slope, mV decade^−1^	52.51 ± 0.20	51.51 ± 0.25
Intercept	480.98	392.98
Correlation coefficient	0.9865	0.9973
Percent recovery (%) ± SD	98.97 ± 0.53	98.62 ± 0.74
Detection limit (mol L^−1^)	9.81 × 10^−8^	1.2 × 10^−7^
Working pH range	3–6	2–7
Life time, day	82	35
RSD (%)	1.54	1.69
Accuracy (%)	99.27	98.85
Precision (%)	0.98	1.88

**Table 3:** Determination of ketotifen fumarate in pure solutions and pharmaceutical preparations using CPE and PVC sensors.

		CPE				PVC			
Taken (mg)		Direct method		Standard addition method		Direct method		Standard addition method	
		Recovery %	RSD %	Recovery %	RSD %	Recovery %	RSD %	Recovery %	RSD %
Pure KTF	4.255	98.93	0.74	98.54	1.77	98.50	0.80	99.02	2.25
	12.77	97.97	0.97	97.89	1.54	98.07	1.46	98.56	1.35
	21.28	98.96	1.14	99.02	0.99	99.18	1.42	97.86	1.87
	29.79	97.83	1.73	99.42	0.83	98.79	1.36	98.22	1.76
Zaditen tablet (1 mg/tablet)	4.26	98.90	0.98	99.00	1.65	97.48	2.08	99.16	1.64

**Table 4:** Determination of KTF in spiked human urine using CPE and PVC membrane electrodes.

Sample	Statistical parameters	CPE			PVC		
		Direct method	Calibration graphs	Standard addition method	Direct method	Calibration graphs	Standard addition method
Human urine	Mean recovery (%)	98.43	98.28	97.89	98.73	98.09	98.17
	*N*	4	4	4	4	4	4
	Variance	0.847	0.748	0.657	0.454	0.607	0.439
	SD	0.752	0.455	0.768	0.859	0.559	0.947
	SE	0.398	0.695	0.836	0.693	0.465	0.551
	RSD (%)	0.849	0.479	0.790	0.906	0.573	0.994
