# Erratum to “Potentiometric Determination of Ketotifen Fumarate in Pharmaceutical Preparations and Urine Using Carbon Paste and PVC Membrane Selective Electrodes”

**DOI:** 10.1155/2012/154907

**Published:** 2012-05-03

**Authors:** Eman Y. Z. Frag, Gehad G. Mohamed, Mohamed M. Khalil, Mohammad M. A. Hwehy

**Affiliations:** ^1^Chemistry Department, Faculty of Science, Cairo University, Giza 12613, Egypt; ^2^Chemistry Department, Faculty of Science, Beni-Suef University, Beni Suef 62111, Egypt

The authors would like to make the following corrections: in Section 1. *Introduction *(Page 1. Line 6), [3, 4] should be changed into [1, 2]; in the same section (Page 1, Line 12), [1, 2, 5] should be changed into [3–5]. 

Also, in Section 2.1. *Reagents* (Page 2, Line 11), potassium tetraphenylborate (KTPB) should be changed into sodium tetraphenylborate (NaTPB); in Section 2.8. *Analysis of Pharmaceutical Samples* (Page 2, Line 7), KTPB should be changed into NaTPB.

